# Collecting size-selectivity data for Antarctic krill (*Euphausia superba*) with a trawl independent towing rig

**DOI:** 10.1371/journal.pone.0202027

**Published:** 2018-08-10

**Authors:** Ludvig A. Krag, Bjørn A. Krafft, Arill Engås, Bent Herrmann

**Affiliations:** 1 Technical University of Denmark, Hirtshals, Denmark; 2 Institute of Marine Research, Bergen, Norway; 3 SINTEF, Hirtshals, Denmark; Fred Hutchinson Cancer Research Center, UNITED STATES

## Abstract

For the development of efficient trawls to minimize catch loss, escape mortality and potential negative ecosystem impacts from the fishery, the understanding about trawl selectivity processes are crucial. Small crustaceans are regarded as being less motile than most fish species. Crustaceans also display low levels of active avoidance from trawl netting, which in turn may cause direct contact with netting on multiple occasions on their passage towards the codend increasing the probability for escapement. Full-scaled experiments to estimate gear selectivity are highly resource demanding and are highly technically challenging for several types of fisheries. In this study, we developed and tested a trawl-independent towed-rig construction designed to investigate size selectivity of Antarctic krill (*Euphausia superba*). The results indicate that valid selectivity estimates can be obtained using this method, but due to the small sample size, results are inconclusive. However, the findings of the current study show a potential for developing easier and more cost-effective ways of investigating and estimating size selectivity of Antarctic krill and other small crustacean species in trawls.

## Introduction

Many species of fish, crustaceans and other organisms are targeted by trawls in fisheries around the world. Fish are highly motile organisms and during the towing process, several species display “herding behavior” by avoiding the netting of the trawl body. Fish are subject to size selectivity, often escaping through the mesh in the codend of trawl nets [1–4]. In contrast, it has been found that 40% of Norway lobsters (*Nephrops norvegicus)* that entered a Norway lobster-trawl managed to escape through the trawl body and 10% through the codend meshes [5]. Comparable results are reported from fisheries of smaller crustaceans like deep water shrimp (*Pandalus borealis*) [6] and brown shrimp (*Crangon crangon*) [7,8]. Smaller crustaceans display low swimming speeds and little active net avoidance, hence enabling contact with the netting throughout the length of the trawl [5]. The capture of these crustaceans resembles more a sieving process due to their low active net avoidance behavior in addition to limited swimming capabilities relative to the towing speed.

Antarctic krill (*Euphausia superba*) is targeted by using pelagic trawls spread with otter boards where the catch is hauled onboard, or by using pelagic beam trawls with a pumping system that continuously supply the catch onboard [9]. The trawls used are typically low tapered and small meshed constructions, often measuring 200 m of length. Krag et al. [10] estimated the size selectivity for krill in different mesh sizes, and mesh opening angles. Based on these results and the selectivity patterns found for crustaceans in general, it is expected that the size selectivity of krill occurs throughout the entire length of the trawl. Krag et al. [10] also demonstrated that krill escape trawl meshes head first and relatively perpendicular to the netting wall. This suggests that individual krill can either orientate themselves in relation to the trawl netting to escape or alternatively, display low swimming capability in relation to the towing speed, making the size selection route a more passive sieving process. In this system, the krill encounter the mesh frequently while moving through the trawl, increasing likelihood of encountering the mesh at an optimal orientation for escape [10].

Krafft and Krag [9] and Krafft et al. [11] found that the escape mortality for krill in small meshed pelagic beam trawls is low (4.4 ± 4.4%), in relation to e.g. many pelagic fish species. However, for developing efficient trawls to minimize catch loss, escape mortality and potential negative ecosystem impacts from the fishery, an increased understanding about the selectivity process for krill is crucial. Previous size selection studies of crustaceans in trawls have employed collection bags mounted on selected spots along the trawl netting on hard-tapered trawl designs, like Norway lobster trawls [5] and demersal shrimp trawls [6–8]. Trawls with hard-tapering is required to ensure that collection bags remain open and capable of collecting escapees during the fishing process. Initial experiments have been performed using small meshed collecting bags on commercial krill trawls, with the results indicating that collecting bags are unfit to catch escapees in these low tapered trawls [12]. Also, size selectivity studies carried out on-board trawlers using continuous pumping systems, which allow their trawls to be deployed at fishing depth for several days or weeks, exclude the use of traditional sampling methods to estimate selectivity (see [3]).

To assess the size selection process of krill in commercial trawl netting, we designed and tested the potential for using a trawl independent multi-compartment towing-rig. The rig was designed to quantify the size selection process in detail and should be simple to operate compared to a full-scale trawl.

## Materials and methods

### Ethical statement

This study did not involve endangered or protected species. Experimental fishing was conducted on board a Norwegian commercial trawler. No permit was required to conduct the study on invertebrates. Field permit was granted by CCAMLR (Commission for the Conservation of Antarctic Marine Living Resources).

Experimental fishing with the towing-rig was carried out off the coast of South Orkney Islands, Antarctica (60°35′S, 45°30′W), during February 2013, with the commercial krill trawler FV *Saga Sea* (Loa 96 m, 6000 Hp.). When a krill swarm was registered on the vessels echo sounder (Simrad EK60) the rig was deployed at sea using one of the vessels 35 mm main towing wires with the purpose of towing into the swarm. Towing speed was set to 2.5 knots following commercial practice.

### Design of the towing-rig

**Fig 1:**
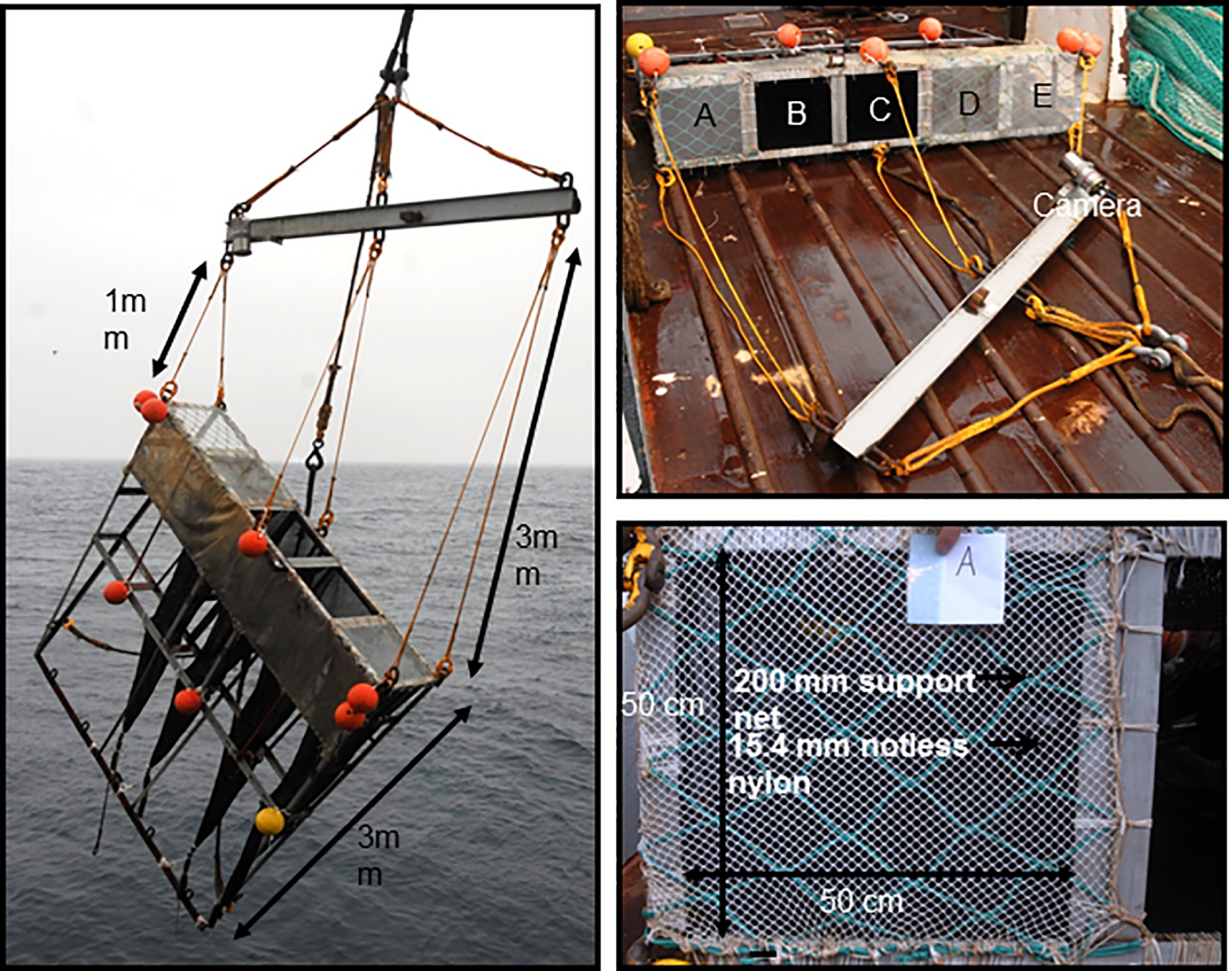
Trawl independent towing-rig for estimating size selectivity of krill.

The towing-rig, measuring 3.0 × 3.0 × 0.6 m was constructed with a steel frame. The forward part of the towing-rig is made up by five equal sized compartments (A-E) (Fig 1). Each compartment has a front opening measuring 50 × 50 cm (Fig 1). The four compartments A-D each had a 3 m long collecting bag made of 7 mm standard survey trawl netting [9,11,13] to collect krill entering each of these four compartments. The entrance to compartments B and C were open. The entrances to compartments A, D and E were covered with a knotless nylon net (commonly used for commercial krill fishing), with 15.4 mm mesh size, supported by a 200 mm double 4 mm PE netting stretched tightly underneath to avoid concavity (Fig 1) The 15.4 mm netting, covering the compartment entrances was stretched and mounted to represent similar and realistic mesh opening angles, comparable to the opening angle values obtained during commercial fishing (see [10]).

Each collecting bag was attached to the rear of the towing-rig and had a nylon ring with a diameter of 25 cm, 1 m from the individual compartments cod-lines to ensure full opening of the collecting bags during deployment (Fig 1). The rig was towed using a 6 m long H-steel beam weighing 200 kg. Crowfeet of different lengths could be attached to the beam to control the towing angle of the rig. We attached three crowfeet, measuring 1 m, 2 m and 3 m, respectively, resulting in a towing angle of about 40 degrees relative to the towing direction of the vessel (Fig 1). In the initial test of the towing-rig a high towing angle in comparison to the tapering of the trawl net relative to the towing angle in the commercial trawls was chosen, to be able to monitor both the performance of the rig and individual krill interacting with the different compartments. A camera (Sony mini DV Digital Handycam) was mounted on the towing beam overviewing the compartments the towing-rig (Fig 1) during all hauls. No artificial light was used during under water video recording as light may bias the natural behavior of krill [11].

During fishing, compartment E was in the forward most position and compartment A in the aft most position (Fig 1). The towing frame was constructed to estimate selectivity for the commercially used mesh sizes by comparing catches in different compartments and comparing those results with experimental trawl based selectivity estimates to give an evaluation of the use of such trawl independent towing-rigs. The arrangement of compartments in the towing rig was further designed with the aim of investigating the interaction between krill and the trawl netting during the size selectivity process.

The individual krill in each haul were measured (± 1 mm) from the anterior margin of the eye to the tip of telson excluding the setae, following to the “Discovery method” used in Marr [14].

### Analysis of selectivity

In most studies investigating the size selection of small crustaceans in diamond mesh codends for trawls, a standard logistic model has been found to be sufficiently flexible to describe the process [15]. A standard logistic size selection model has also been found to be able to describe the size selection of Antarctic krill in trawls made of diamond mesh netting [10]. It was also expected that the standard logistic model would be able to describe the size selection of Antarctic krill in diamond netting panels during the towing of the rig. Therefore, this model was initially used with the intention of only considering other size selection models if the logistic model did not describe the experimental data sufficiently well. The standard logistic model is fully defined by the two parameters L50 (length of organism with 50% probability of being retained) and SR (= L75–L25):

$$r_{logistic}(l,L50,SR)=\frac{exp(ln(9)\times (l-L50)/SR)}{1.0+exp(ln(9)\times (l-L50)/SR)}$$ (1)

where *l* is the length of the Antarctic krill.

Let *nA*_*l*_ and *nB*_*l*_ be the number of krill in length class *l* that are collected in compartments A and B, respectively during the sampling with the towing rig. The experimental fraction *RA*_*l*_ observed in compartment A of the total (*nA*_*l*_ *+ nB*_*l*_) is:

$$RA_{l}=\frac{nA_{l}}{nA_{l}+nB_{l}}$$ (2)

Let *n*_*l*_ be the total number of krill in length class *l* making contact with either the entrance of compartment A or B during the sampling the towing rig and let *SP* be the assumed size independent fraction of those that make contact with the entrance of compartment A. Furthermore, let *r(l)* be the size selection in the netting covering the entrance to compartment A. The expected number of krill in length class *l* to be observed in compartments A and B, respectively will then be:

$$\begin{array}{c} nA_{l}=SP\times n_{l}\times (1.0-r(l))\\ nB_{l}=(1.0-SP)\times n_{l} \end{array}$$ (3)

Inserting (2) into (1) leads to:

$$RA_{l}=\frac{SP\times n_{l}\times (1.0-r(l))}{SP\times n_{l}\times (1.0-r(l))+(1.0-SP)\times n_{l}}=\frac{SP-SP\times r(l)}{1.0-SP\times r(l)}$$ (4)

When considering only compartments A and B the data are binomial since observed krill that are not in compartment A will be in compartment B. The theoretical expressions for the expected size dependent catch sharing *RA(l)* and *RB(l)* between compartment A and B will based on (3):

$$\begin{array}{c} RA(l)=\frac{SP-SP\times r(l)}{1.0-SP\times r(l)}\\ RB(l)=1.0-RA(l)=\frac{1.0-SP}{1.0-SP\times r(l)} \end{array}$$ (5)

Assuming a logistic size selection model *r*_*logistic*_*(L50*, *SR*,*l)* for the size selection in the netting covering compartment A and that the fate of individual krill are independent of each other, the sampled two-compartment binomial count data for number krill of *nA*_*l*_ and *nB*_*l*_ in each length class *l* found in compartments A and B, respectively can be used to estimate the selection parameters *L50* and *SR* by maximizing the likelihood for the observed data. Technically this is done by minimizing the negated natural logarithm *ln ()* of the likelihood function with respect to *L50*, *SR* and *SP*. It can be expressed as:

$$-\sum_{l}\left\{nA_{l}\times ln(\frac{SP-SP\times r_{logistic}(L50,SR,l)}{1.0-SP\times r_{logistic}(L50,SR,l)})+nB_{l}\times ln(\frac{1.0-SP}{1.0-SP\times r_{logistic}(L50,SR,l)})\right\}$$ (6)

where the summation is over length classes *l* in the sampled krill data.

Estimating size selection in trawl netting based on minimizing (6) with respect to parameters L50, SR and SP has a similar structure to the model applied when estimating trawl size selectivity based on paired-gear data where a non-size selective control codend is towed parallel or alternately with the test codend subject to investigation [3]. Size selection data collected using paired-gear are analysed following the SELECT method [16] which is based on modelling the observed catch data in form of sharing between the test and control codend. In the current study, the SELECT method not only provided estimates the value of selection parameters L50 and SR but also the entry sharing ratio SP often called the “split parameter”. The difference between our method and the paired-gear data collection method is that it samples the individuals that are retained in the tested netting (test codend) whereas our design samples the individuals that “escape” through the tested netting (collecting bag for compartment A). This results in formula (6) differing slightly from the formula minimized in the estimation of selection parameters when this is based on the SELECT method for paired-gear data. However, both methods include sampling an estimate for the size structure of the population available for size selection, in our case with the catch collected in compartment B and in case of the paired-gear method with the catch in the non-selective control codend. We name the new method described above based on comparing catches in compartments A and B by on using (6) to estimate the netting size selection “The Inverse paired-compartment method”.

The collected data were pooled for the nine hauls carried out with the towing rig prior to conducting the analyses with (6) to obtain the average size selection estimation for the netting by using the inverse paired-compartment method. The selectivity data were analysed using the analysis tool SELNET (SELection in trawl NETting; [17]). Evaluating the ability of the model to describe the observed data sufficiently well was based on inspecting the fit statistics, i.e. the p-value and the model deviance versus the degrees of freedom (DOF), following the procedures described by Wileman et al. [3]. The p-value expresses the likelihood to obtain at least as big a discrepancy between the fitted model and the observed experimental data by coincidence. In case of a poor fit [p-value being <0.05; deviance being >> degrees of freedom), the residuals and the ability of the model formulas (3) to follow the main trends in the experimental catch sharing rate (formulas (1)] were inspected to determine whether the poor result was due to structural problems when describing the experimental data using the model or over-dispersion in the data [3]. Confidence intervals for the selection curves and the selection parameters where obtained based on the bootstrap methods implemented in SELNET using 1000 bootstrap repetitions to obtain the Efron percentile 95% confidence limits [18,19]. The method applied accounts both for within and between haul variation in the selection process by resampling over hauls in an outer bootstrapping loop and over length classes in selected hauls in an inner bootstrapping loop [15, 17].

### Alternative size selection estimations based on the towing rig

In the previous section we described how the size selection in the netting could be estimated based on comparing the catches in the collecting bags from compartments A and B. All compartments were monitored during fishing to determine if individuals were transported from one compartment to the next. Assuming that the transportation of krill along the netting panels during sampling is negligible, size selection can be estimated by the same approach as described in the previous section, but comparing other pairs of compartment catches other than A vs. B. Assuming negligible transport of krill along the netting on compartment D to compartment C we can use compartment C to sample the entry population instead of compartment B. Similarly, by comparing compartment E with compartment D we can use compartment D instead of A to sample the net selected population. Therefore, assuming negligible krill transportation along the netting panels in the towing rig, the following alternative compartment pairs can be used to estimate the netting size selection using the inverse paired-compart method described in the last section:

- Compartment A versus compartment C
- Compartment D versus compartment B
- Compartment D versus compartment C
- Compartment A+D versus compartment B+C

### Comparing size selectivity for the towing rig to previous obtained for trawl

To infer if the estimated size selectivity by using the towing rig differed from the size selectivity obtained with a trawl with same mesh size (Krag et al. [10]), the difference in the length-dependent retention probability Δ*r*(*l*) was estimated:

$$\Delta r(l)=r_{rig}(l)-r_{trawl}(l)$$ (7)

where *r*_*rig*_(*l*) is the size selection curve obtained for the towing rig for each of the assessment methods: compartment A versus compartment B, compartment A versus compartment C, compartment D versus compartment B, compartment D versus compartment C and compartment A+D versus compartment B+C. *r*_*trawl*_(*l*) is the size selectivity curve obtained by Krag et al. [10]. The 95% confidence intervals for Δ*r*(*l*) were obtained based on the two bootstrap population results (1000 bootstrap repetitions in each) for *r*_*trawl*_(*l*) and *r*_*rig*_(*l*), respectively. As they are obtained independently from each other, a new bootstrap population of results for Δ*r*(*l*) was created using:

$${\Delta r(l)}_{i}={r_{rig}(l)}_{i}-{r_{trawl}(l)}_{i}\;i\in [1\ldots 1000]$$ (8)

where *i* denotes the bootstrap repetition index. As resampling was random and independent for both groups of results, it is valid to generate the bootstrap population of results for the difference based on (8) using two independently generated bootstrap files [20, 21]. Based on the bootstrap population, Efron 95% percentile confidence limits were obtained for Δ*r*(*l*) as described above.

## Results

The towing-rig was simple to operate and fast to deploy, retrieve and collect catch from. It however, proved challenging to target smaller acoustic registrations of krill, especially in water deeper than 100 m as there was no depth sensor giving live data to the bridge on the towing-rig that could indicate the rigs position relative to the targeted swarm of krill.

**Fig 2:**
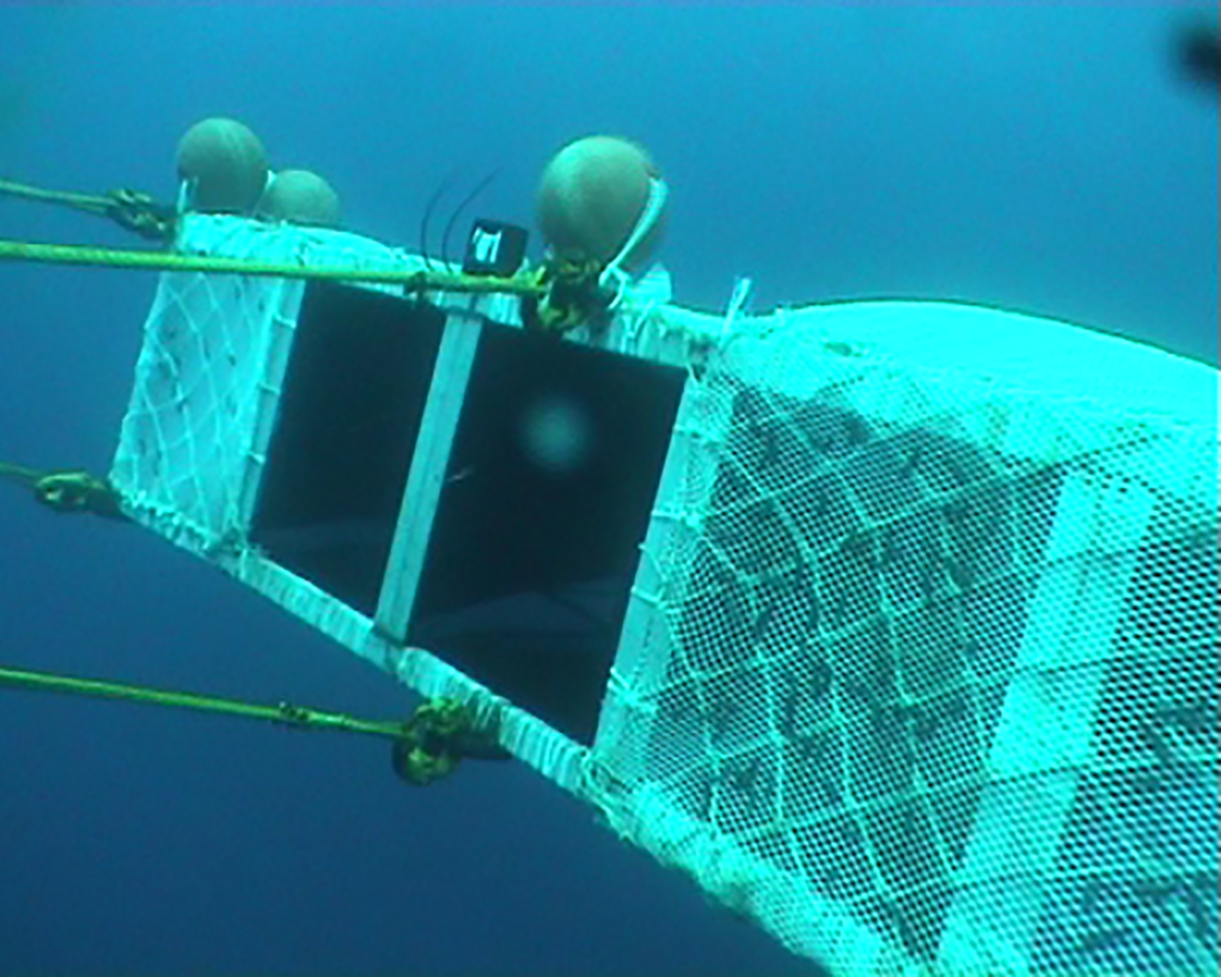
Picture of the towing-rig grabbed from underwater video during fishing. Compartment A is in the left side and compartment E is partly visible in the right of the image. The camera overlooking the towing-rig is mounted on the towing beam (see Fig 1) in an angle which may give a misleading impression of the actual towing angle of the towing-rig. Notice krill on netting on compartments E, D and A covered with netting.

A total of nine hauls were made with the towing rig. The operational conditions for each haul are given in Table 1. Analysis of the UV film show that the towing-rig moves stably and in a horizontal position during towing (Fig 2). However, detailed inspections of images show that some individuals that encountered compartments A, D and E were lost over the towing rig indicating that it had a small backwards tilt. No individuals were observed to roll from one compartment to the next. These observations were based on the data from only two out of the nine hauls, which had enough light for quantitative interpretations of the video recordings.

**Table 1:** Operational conditions.

					
	Trawling (GMT)		Duration	Depth	Catch
Haul no.	Start	End	min.	m.	no.
1	23:08	23:27	19	180	170
2	00:37	00:39	2	5	219
3	11:35	12:05	30	100	17
4	15:20	15:55	35	72	96
5	07:13	07:35	22	72	66
6	12:36	13:10	34	156	60
7	13:28	13:42	14	161	16
8	14:02	14:36	34	155	19
9	14:46	15:15	29	140	13

**Fig 3:**
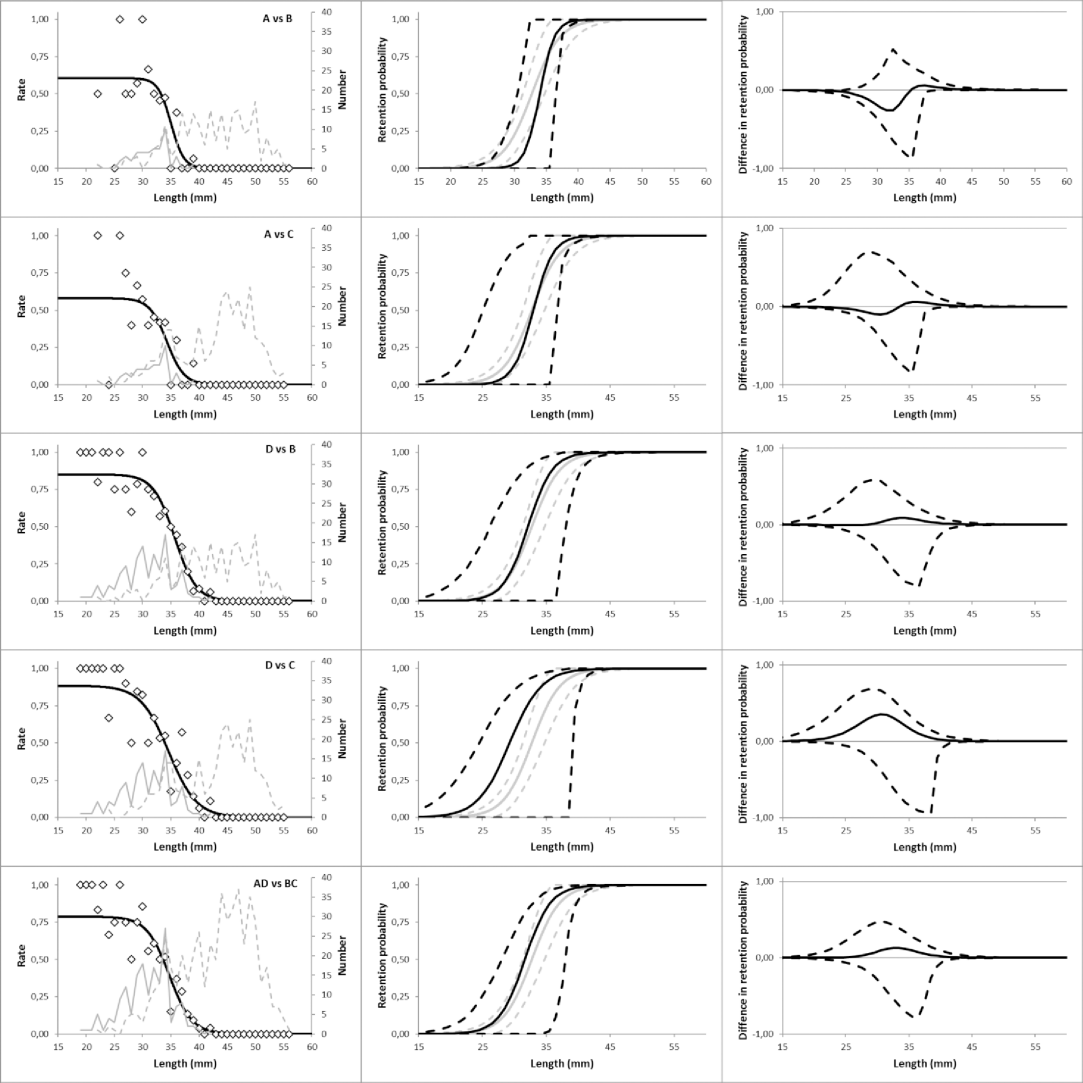
Modelled catch sharing curves. (left column), size selectivity curves (center column) for paired estimation and difference in size selectivity compared to Krag et al. [10] (right column) based on (from top) compartments: A vs. B, A vs. C, D vs. B, D vs. C and A+D vs. B+C, respectively. Diamond marks represent experimental catch sharing rates while the black curves represent the modelled catch sharing curves (in left column). In the left column, the grey curves represent the population of krill caught in compartments A and D (solid curves) and compartments B and C (broken curves). In the center column, the broken curves represent the 95% confidence bands for the size selectivity curves with the grey curves representing results from Krag et al. [10].

Size selectivity results were also obtained based on similar paired comparisons between the other compartments and combination of compartments, e.g. D vs. B, A vs. C, D vs. C and between A+D vs. C+D and A+B vs. D+C (Fig 3). The intercept of the estimated paired curve with the y-axis indicates the estimated split or the proportion of individuals entering compartments A and B, respectively (Fig 3). The estimated split value is around 0.6 indicating that more individuals were in contact with compartment A than B. The paired curved and the selectivity curves for these comparisons are given in Fig 3. Comparing B and D, provided a split value estimate of about 0.8. The estimated split values indicate that more individuals encountered compartment D than compartment B. Similar high split values, above 0.5, were observed for the remaining comparisons (Fig 3), suggesting that the assumption of equal proportions of individuals encountering each compartment may be invalid.

The estimated selection curve for all pairwise comparisons seems well described by a logit model as supported by fit statistics (Table 2). However, in general results exhibited under-dispersion as deviance in all cases was lower than DOF, probably due to sparsity of data. Especially for compartment A versus B and for compartment D versus B is obtained value for the deviance much smaller than DOF. The sparsity in data is also reflected in the wide confidence bands in the estimated size selectivity (Fig 3, center column). The selectivity curves are compared with an experimentally obtained selectivity curve for the same mesh sizes found in Krag et al. [10] (Fig 3, center and right column). Based on the obtained results there is no evidence of difference in size selection between the two sets of estimates as the confidence bands for difference in size selection (Fig 3, right column) contain 0.0 for all sizes of krill. However, for a wide range of length classes (28–36 mm) are the confidence bands for Δ*r*(*l*) wide which imply that the obtained results neither can rule out a considerable difference in size selection between results obtained with the towing rig compared to by Krag et al. [10]. Therefore, results are rather inconclusive based on the collected data set with the towing rig, but the analysis of the data demonstrates the method and the potential with it. The parameter estimates (L50 and SR) from Krag et al. [10] and the different compartment comparisons in the towing-rig are quite similar, especially the L50 estimates ranging from 29.25 to 33.96 mm (Table 2). However, as in line with above wide confidence bands for the towing-rig estimates for L50 and SR makes the comparison with estimated values in Krag et al. [10] inconclusive (Fig 3, Table 2), which is probably due to the relatively low number of individuals caught in the current study.

**Table 2:** Estimated selectivity parameters for the five different compartment comparisons. DOF denotes degree of freedom. Values in brackets represent 95% confidence limits.

	Compartment				
	A vs. B	A vs. C	D vs. B	D vs. C	A+D vs. B+C
**L50 (mm)**	33.96 (30.46–36.50)	32.96 (25.09–36.52)	32.11 (26.21–38.07)	29.25 (24.52–39.15)	31.64 (28.00–37.93)
**SR (mm)**	2.51 (0.10–3.56)	3.38 (0.10–5.19)	4.16 (0.10–6.99)	5.46 (0.10–7.37)	4.40 (1.08–6.31)
**SP**	0.61 (0.13–0.95)	0.58 (0.13–0.95)	0.85 (0.49–0.95)	0.88 (0.46–0.95)	0.79 (0.48–0.91)
**P-value**	0.96	0.79	0.99	0.65	0.90
**Deviance**	17.91	22.65	17.81	30.23	24.73
**DOF**	30	29	35	34	35
**L50 mm (Krag et al.) [10]**	32.72 (30.98–34.46)				
**SR (mm) Krag et al. [10]**	4.85 (2.75–6.95)				

## Discussion

We tested the trawl independent towing-rig to investigate if such constructions can provide valid size selectivity estimates for the commercially targeted Antarctic krill. Because of the relatively small towing rig sample size, also reflected by the wide confidence bands for the estimated size selection curves, no firm conclusions about the actual selection process could be drawn. Unfortunately, it was not possible to perform additional tows with the rig during the time we had available for these experiments while at sea. However, the paired gear comparisons resulted in selectivity curves and parameter estimates (L50 and SR) that was similar to the selectivity estimates obtained by Krag et al. [10], which was based on full scaled trawl krill trials using the same mesh size. The results from this study are inconclusive regarding the ability to replace trawl experiments by experiments with the towing rig, but further experiments based on larger samples with the towing rig may prove its applicability. The towing-rig based method including data treatment and modelling work developed herein, may represent an alternative tool to estimate size selectivity of krill to complement or even replace traditional trawl based selectivity experiments for smaller crustaceans [3]. The method is time and cost efficient and does not have the same logistical requirements as traditional trawl trials. If first proven valid for one mesh size and type the system, it can also likely be modified to produce selectivity estimates for a diverse range of mesh sizes, shapes and tapering angles.

The underwater video recordings were useful for observing the behavior of the towing-rig and the interactions of individual krill with the rig during towing. It showed that the towing-rig had a small backwards tilt, resulting in some individuals escaping above the top edge ridge. It also indicated a small concavity in the compartments covered with the 15.4 mm netting during fishing, despite the stretched underlying 200 mm diamond netting intended to prevent this. Small compartment covered with netting may behave different from large netting sheets in commercial trawls. For future studies, these issues can be resolved with some simple adjustments to the rig. No transportation of individuals from compartment to compartment was observed from our underwater video recordings. Another advantage of such small and rigid towing systems is that they are easy to fully monitor with a wide angle lensed camera, allowing the interactions of individual organisms with the net under different towing conditions to be studied in detail.

The results of this study using the trawl independent towing rig system shows that there is potential to improve the experimental design to gain new knowledge, regarding the interactions of different crustacean species with different meshes and towing angles during the selectivity process. The towing angle applied in the current study was higher compared to the tapering in a traditional commercial krill trawl. Krag et al. [10] described the theoretical effect of the angle of the netting relative to the towing direction where a steeper angle led to higher selective potential. Further development of such towing-rigs could be aimed at designing a system where the towing angle is adjustable; including commercial towing angles, which also ensures that all escapees can be collected in open compartments without the risk of masking the netting. Sensors capable of documenting and continuously monitoring the systems performance e.g. vertical and horizontal stability, towing angle, towing speed and depth in combination with full camera surveillance during operation are necessary to test the applicability of this system. Norway lobster surveys in the North Sea are partly conducted using towed under water sledges equipped with a camera, LED lights and different sensors. These systems are towed using a steel enforced Coaxial cable allowing live-streaming from the sledge to the vessel. Similar cables allowing live monitoring of the process and the performance of the towing-rig could enhance the development and use of such systems and make the operation independent of the main towing wires on board fishing vessels. To ensure sufficient catch levels for firm statistical analysis, depth data from the towing rig need to be available in real time on the vessels bridge to enable maneuvering of the towing rig to the echogram registrations of krill.

The current study indicates the potential for studying size selectivity of other small crustacean species. In shrimp trawl fisheries, e.g. *Crangon* fisheries, the size selectivity of different mesh sizes, mesh types or grid systems with different bar spacing could potentially be explored using a trawl independent towing-rig. It is however clear that such systems need to be customized for each case or fishery. In the *Crangon* fishery, which is a demersal beam trawl fishery [8] the towing-rig could be constructed with a sledge to be towed along the seabed. Avoidance behavior in relation to trawling could also be examined, by designing various size compartments in the towing-rig and examining the catch for differences in mean sizes as a proxy for active swimming/avoidance behavior. Active avoidance could then be indicated by lower mean sizes in the smaller compartment. It is however, important that selectivity estimates provided by devices that are constructed different from what they are intended to describe, always are compared or inter-calibrated with experimental trawl based results, e.g. for one mesh size to validate the devices ability to describe the process in quest.
